# Extranodal MALT Lymphoma of the Right Triceps Muscle following Influenza Vaccine Injection: A Rare Case with an Interesting Presentation

**DOI:** 10.5402/2011/617293

**Published:** 2011-06-09

**Authors:** S. M. Edwards-Bennett, D. Straus, E. A. Athanasian, J. Yahalom

**Affiliations:** ^1^Department of Radiation Oncology, H. Lee, Moffitt Cancer Center & Research Institute, 12902 Magnolia Drive, Tampa, FL 33612, USA; ^2^Departments of Medicine and Radiation Oncology, Memorial Sloan Kettering Cancer Center, 1275 York Avenue, New York, NY 10021, USA; ^3^Department of Orthopedic Surgery, Hand and Upper Extremity Service, Hospital for Special Surgery, 535 East 70th Street, New York, NY 10021, USA

## Abstract

The study describes a case of a 67-year-old female who developed a Stage I E marginal zone lymphoma of the right triceps muscle 1 month after influenza vaccination at the same site. She was treated with single modality, involved field radiation therapy (IFRT) to 4000 cGy in 20 fractions with excellent response and no evidence of disease after one year followup.

## 1. Introduction

Extranodal marginal zone lymphomas (ENMZLs) constitute 7-8% of all B-cell lymphomas [1]. They most commonly occur in the stomach but can present in the salivary glands, Waldeyer's ring, large and small intestine, thyroid, eye (orbit and conjunctiva), lung, breast, and skin [2]. Involvement of the muscle is very rare and accounts for less than 1% of all mucosa-associated lymphoid tissue (MALT) lymphomas [3]. 

There is some evidence to suggest that MALT lymphoma develops as a result of prolonged antigenic stimulation by autoantigens and/or microbial pathogens. For example, MALT lymphoma of the thyroid gland develops in patients with Hashimoto's thyroiditis [4] and in salivary glands of patients with Sjögren's syndrome [5]. The most common association is, however, Helicobacter pylori infection and MALT lymphoma of the stomach [6].

The case presented herein is a rare case of a 67-year-old female who developed MALT lymphoma of the right triceps muscle after the influenza vaccination at the same site one month earlier.

## 2. Case Report

**Figure 1:**
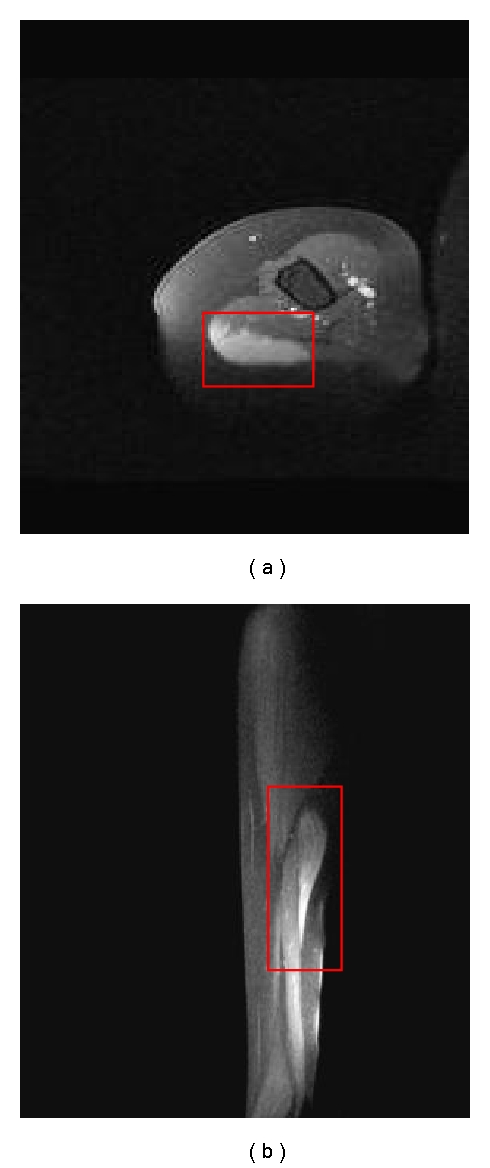
Pretreatment axial (a) and sagittal (b) postgadolinium contrast MRI of the right upper arm with enhancing mass in the posterior lateral aspect of the long head of the right triceps muscle outlined in red.

A 67-year-old female received influenza vaccination in summer, 2009. One month later she developed pain and lump in the right arm reportedly in the same area that was vaccinated. Upon initial presentation her symptoms were presumed to be secondary to a torn triceps muscle. She was thus referred to an orthopedist who recommended imaging workup. Magnetic resonance imaging (MRI) of the right upper arm showed a 4.3 × 1.1 × 7.5 cm enhancing mass in the posterior lateral aspect of the long head of the right triceps muscle (Figure 1).

Core biopsy revealed low grade lymphoma of small B-cell type. Additional tissue sampling was recommended for further classification and immunophenotyping. She underwent incisional biopsy that demonstrated B-cell-type lymphoma infiltrating the skeletal muscle. Immunohistochemical staining was positive for CD 20, BCL2 and pax-5 and negative for CD3, CD5, CD10, CD 23, BCL 6, and cyclin D1, consistent with marginal zone lymphoma. Bone marrow biopsy was negative. Lactate dehydrogenase (LDH) was within normal limits. She was therefore staged I E with International Prognostic Index (IPI) score of 1 by virtue of her age. 

After multidisciplinary consultations, a course of definitive radiation therapy was recommended. She underwent CT simulation in the supine position with alpha-cradle for immobilization. The target lesion was contoured as the gross tumor volume (GTV). The GTV was expanded by 2 cm to create the planned treatment volume (PTV).

She received 40 Gy in 20 fractions with 6 MV photons utilizing 3D conformal radiation therapy. She tolerated the treatment well without any unexpected side effects.

Physical examination at 1 and 6 months and 1 year after treatment revealed no palpable residual mass, swelling or skin erythema, or range of motion deficits.

**Figure 2:**
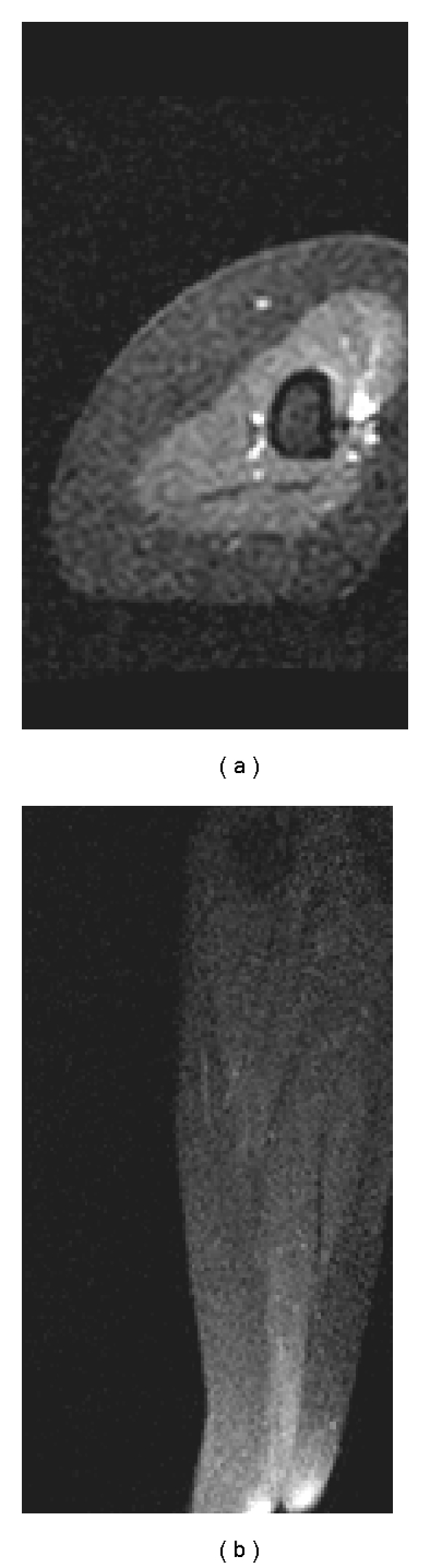
Posttreatment axial (a) and sagittal (b) postgadolinium contrast MRI of the right upper arm demonstrating resolution after a course of IFRT to 40 Gy.

6-month posttreatment MRI of the right upper arm demonstrated excellent response with no evidence of residual disease (Figure 2). Computerized tomography of the chest, abdomen and pelvis (CT CAP) at 1 year showed no evidence of disease.

## 3. Discussion

Extranodal MALT lymphoma comprises 7-8% of non-Hodgkin lymphoma (NHL) and most commonly involves the stomach. [1] As such, the treatment of stomach MALT with involved field radiation therapy has been well established, yielding excellent outcomes with over 90% local control in most series [7–9].

Nongastric marginal zone lymphoma is less common, and treatment of this entity is much less defined in the literature. In a large International Extranodal Lymphoma Study Group (IELSG) series of 180 patients with Nongastric MALT, a variety of treatment approaches were employed, including chemotherapy, surgery or radiation alone, or in combination [3].

Even rarer is Nongastric marginal zone lymphoma in the skeletal muscle, so much so that the large Nongastric MALT series, published by the IELSG, did not include any patients with marginal zone lymphoma of the skeletal muscle [3]. To our knowledge, only two cases of ENMZL of the skeletal muscle have been reported [10, 11]. Gill et al. [10] reported a case of stage IV marginal zone lymphoma of the right forearm in the distal biceps and proximal triceps muscles with pulmonary metastases that was treated initially with 6 cycles of standard dose cyclophosphamide, doxorubicin, oncovin and prednisone (CHOP), radiation therapy for locally recurrent disease 4 years later, and finally chemotherapy for a second recurrence. That case may lend support to the claim that primary skeletal lymphoma tends to be more aggressive, present late stage, and portend a poor prognosis [10, 12]. However, our patient presented with early stage (I E) primary skeletal muscle marginal zone lymphoma and an IPI score of 1. 

It was quite interesting that our patient developed MALT in the right triceps muscle a few weeks after receiving influenza vaccination at the same site. There is mounting evidence supporting the association between antigenic stimulation and/or microbial pathogens and MALT lymphoma. For example, patients with autoimmune diseases such as Sjögren's and Hashimoto's thyroiditis have an increased risk of MALT of the salivary glands and thyroid, respectively [4, 5]. There is also a strong association between H. pylori infection and stomach MALT, and yet to be confirmed associations between *Chlamydia psittaci* and adnexal MALT lymphoma and hepatitis C virus (HCV) infection with MALT, splenic, and nodal MZL [13, 14]. Whether our patient's triceps MALT was coincidental or related to her influenza vaccination remains undetermined. Nonetheless, she responded well to IFRT alone with no radiological evidence of residual, recurrent, or distant disease.

## 4. Conclusion

The relationship between influenza vaccine injection and the development of MALT lymphoma in this patient is speculative but is, undeniably, an interesting confluence of events. Although the available literature suggests that primary skeletal muscle NHL is associated with poor prognosis, the case presented here suggests that IFRT alone can be an effective treatment for early stage primary skeletal marginal zone lymphoma.
